# In Vitro and In Vivo Screening of Wild Bitter Melon Leaf for Anti-Inflammatory Activity against *Cutibacterium acnes*

**DOI:** 10.3390/molecules25184277

**Published:** 2020-09-18

**Authors:** Lu-Te Chuang, Ya-Hsin Shih, Wen-Cheng Huang, Lie-Chwen Lin, Chin Hsu, Jong-Ho Chyuan, Tsung-Hsien Tsai, Po-Jung Tsai

**Affiliations:** 1 Department of Biotechnology and Pharmaceutical Technology, Yuanpei University of Medical Technology, Hsinchu 300, Taiwan; ltchuang@mail.ypu.edu.tw; 2 Department of Human Development and Family Studies, National Taiwan Normal University, Taipei 106, Taiwan; yayayungchieh@hotmail.com (Y.-H.S.); wencheng7373@gmail.com (W.-C.H.); 3 National Research Institute of Chinese Medicine, Ministry of Health and Welfare, Taipei 112, Taiwan; lclin@nricm.edu.tw; 4 Department of Exercise Health Science, National Taiwan University of Sport, Taichung 404, Taiwan; jeanhsu@ntupes.edu.tw; 5 Hualien District Agricultural Research and Extension Station, Hualien 973, Taiwan; jonghoc@hdares.gov.tw; 6 Department of Dermatology, Taipei Municipal Wan Fang Hospital and Taipei Medical University, Taipei 116, Taiwan; 7 Program of Nutrition Science, School of Life Science, National Taiwan Normal University, Taipei 116, Taiwan

**Keywords:** wild bitter melon (WBM), *Cutibacteriumacnes*, anti-inflammation, β-ionone

## Abstract

*Cutibacterium acnes* (formerly *Propionibacterium acnes*) is a key pathogen involved in the development and progression of acne inflammation. The numerous bioactive properties of wild bitter melon (WBM) leaf extract and their medicinal applications have been recognized for many years. In this study, we examined the suppressive effect of a methanolic extract (ME) of WBM leaf and fractionated components thereof on live *C. acnes*-induced in vitro and in vivo inflammation. Following methanol extraction of WBM leaves, we confirmed anti-inflammatory properties of ME in *C. acnes*-treated human THP-1 monocyte and mouse ear edema models. Using a bioassay-monitored isolation approach and a combination of liquid–liquid extraction and column chromatography, the ME was then separated into *n*-hexane, ethyl acetate, *n*-butanol and water-soluble fractions. The hexane fraction exerted the most potent anti-inflammatory effect, suppressing *C. acnes*-induced interleukin-8 (IL-8) production by 36%. The ethanol-soluble fraction (ESF), which was separated from the *n*-hexane fraction, significantly inhibited *C. acne*s-induced activation of mitogen-activated protein kinase (MAPK)-mediated cellular IL-8 production. Similarly, the ESF protected against *C. acne*s-stimulated mouse ear swelling, as measured by ear thickness (20%) and biopsy weight (23%). Twenty-four compounds in the ESF were identified using gas chromatograph–mass spectrum (GC/MS) analysis. Using co-cultures of *C. acnes* and THP-1 cells, β-ionone, a compound of the ESF, reduced the production of IL-1β and IL-8 up to 40% and 18%, respectively. β-ionone also reduced epidermal microabscess, neutrophilic infiltration and IL-1β expression in mouse ear. We also found evidence of the presence of anti-inflammatory substances in an unfractionated phenolic extract of WBM leaf, and demonstrated that the ESF is a potential anti-inflammatory agent for modulating in vitro and in vivo *C. acnes*-induced inflammatory responses.

## 1. Introduction

Acne vulgaris (acne) is one of the most common and chronic inflammatory skin diseases associated with abnormal keratinization, increasing sebum production, bacterial colonization and inflammation [1]. It is widely accepted that the commensal bacterium *Cutibacterium acnes* (previously named *Propionibacterium acnes*) is involved in the initiation and prolongation of inflammation [1,2]. For example, *C. acnes* produces numerous hydrolytic enzymes, including lipases, proteases and hyaluronidases, that can damage skin [3], and modulates inflammatory responses triggered when monocytes are stimulated by *C. acnes* through the activation of toll-like receptor 2 (TLR2) [1,3]. During the inflammation, pro-inflammatory cytokines secreted from monocytes attract neutrophils, basophils and T cells to the infected pilosebaceous unit, leading to the process of chronic inflammation [4]. Over-expression and excessive secretion of pro-inflammatory cytokines have been highly correlated with the severity of acne in patients [5]. Since anti-cytokine therapies have been applied for various inflammatory diseases [6], identifying new agents that suppress *C. acnes*-induced inflammation might be useful in treating acne vulgaris.

Wild bitter melon (*Momordica charantia* L. var. *abbreviate* Seringe; WBM), a member of the family Cucurbitaceae, is a tropical and subtropical vine that is native to Asia, Africa and the Caribbean region. WBM fruits and leaves are widely consumed on a daily basis, as well as used as folk remedies to treat numerous conditions and symptoms, including relieving metabolic syndromes [7], diabetes [8] and inflammatory responses [9], lowering blood lipid and glucose levels [10] and also for slowing the progression of certain cancers [8]. Like many other plants, WBM fruit and leaves are rich sources of a wide variety carotenoids and polyphenols [11] that may have beneficial applications in humans. For example, WBM fruit extracts inhibited *C. acnes*-induced in vitro or in vivo inflammatory responses [12]. Furthermore, WBM leaf extracts possessed significant antioxidant, cytoprotective and anti-melanogenic activities [13], and suppressed *C. acnes*-induced inflammation [14]. These findings indicate that extracts of WBM contain antioxidant and anti-inflammatory substances that might be used to treat acne vulgaris and perhaps other ailments as well. We therefore explore bioactive components in WBM leaf extracts which might account for anti-inflammatory properties.

To this end, we first used bioassay-guided fractionation methods to isolate and identify active compounds in a methanolic extract (ME) of WBM leaves. Next, using two *C. acnes*-induced models of human THP-1 monocytes and mouse ear edema, we determined the anti-inflammatory effect of WBM leaf extract and its components against *C. acnes*. In addition, mechanisms underlying WBM leaf extract and its components that suppressed inflammation were explored. This study provides new understanding regarding how active ingredients of WBM extracts modulate in vitro and in vivo inflammatory responses.

## 2. Results and Discussion

### 2.1. Effects of ME of WBM Leaf on C. acnes-Induced Cellular IL-8 Production and Mouse Ear Edema

**Figure 1:**
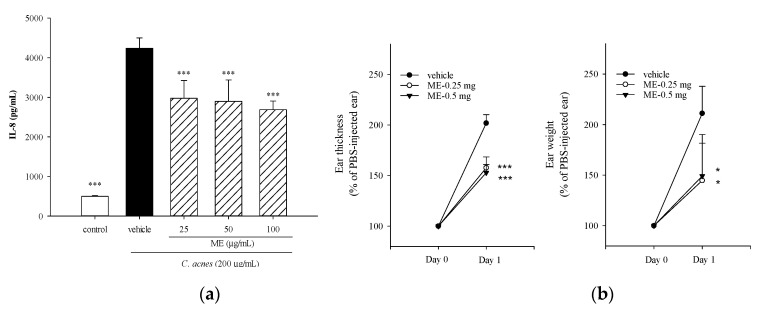
Effects of the crude methanolic extract (ME) of wild bitter melon (WBM) leaf on *C. acnes*-induced IL-8 production in vitro and on ear swelling in mice. THP-1 cells were cultured with DMSO as the negative control, or co-incubated with *C. acnes* (M.O.I. = 75) and different concentrations (25, 50 or 100 μg/mL) of ME for 24 h. The culture supernatants were subsequently collected and analyzed for the IL-8 levels (**a**). In the ear edema mouse model, ME (0.25 or 0.5 mg/site) or vehicle (phosphate-buffered saline; PBS) was intradermally injected, immediately followed by the *C. acnes* injection. The inhibitory effects of ME on *C. acnes*-induced ear swelling were evaluated by measuring the ear thickness and ear biopsy weight (**b**). Each value shows the mean ± SD. Values with different symbols are significantly different from the *C. acnes* control (*C. acnes* alone) at *p* < 0.05 (*) and *p* < 0.001 (***).

To investigate whether incubation of THP-1 cells with the ME of WBM leaf affected cell viability, the culture medium was supplemented with various concentrations (up to 100 μg/mL) of tested samples. No negative effect on cell proliferation was observed when the concentration of WBM leaf ME was 100 μg/mL or less (data not shown). Since IL-8 is a neutrophilic chemokine which plays a critical role in the development of acne vulgaris, we measured the production of IL-8 by THP-1 cells in order to determine whether tested samples suppressed *C. acnes*-induced inflammation. Figure 1a shows that IL-8 production was significantly increased following *C. acnes* stimulation. However, supplementation of the medium with different concentrations of the ME of WBM leaf significantly reduced the production of IL-8 by as much as 36%. Using the *C. acnes*-induced mouse ear edema model, we investigate the in vivo anti-inflammatory effects of the ME of WBM leaf. Inoculation of mouse ears with *C. acnes* increased edema 2.1-fold (*p* < 0.001) relative to the control. WBM leaf ME (0.25 and 0.5 mg) significantly reduced ear swelling as measured by ear thickness (by 25%) and ear disc weight (by 32%) (Figure 1b). These findings indicate that topical injection of the ME of WBM leaf suppresses *C. acnes*-induced inflammation in vivo. Our results are in accordance with previous findings reported by Huang and colleagues, who demonstrated that total phenolic extracts of WBM leaves mitigated inflammation by suppressing *C. acnes*-induced production of cellular cytokines and mouse ear swelling [14]. Furthermore, ethanol/ethyl acetate extract of WBM leaf inhibited heat-killed *P. gingivalis*-induced cytokine production by THP-1 cells [15]. Collectively, these findings document that WBM leaf extracts suppressed bacteria-induced inflammation in vitro and in vivo.

Lu and colleagues previously demonstrated that WBM extracts inhibit the growth of a variety of bacterial species [16]. In the present study, having documented that the ME of WBM leaf reduces cytokine production by virtue of its anti-inflammatory properties, we speculated that the decrease in pro-inflammatory mediator production might be due to the anti-bacterial effect of the ME of WBM leaf. In fact, the results of our antibacterial assay showed that the minimal inhibitory concentration (MIC) of WBM leaf ME was higher than 500 μg/mL; thus, the MIC value was at least 5-fold higher than the highest concentration (100 µg/mL) of WBM leaf ME included in the THP-1 cell culture medium. We conclude, therefore, that the anti-bacterial properties of WBM leaf ME do not contribute to the reduction of *C. acnes*-induced pro-inflammatory cytokine production.

### 2.2. Effects of Four Partitioned Fractions from ME of WBM Leaf on C. acnes-Induced Cellular IL-8 Production

**Figure 2:**
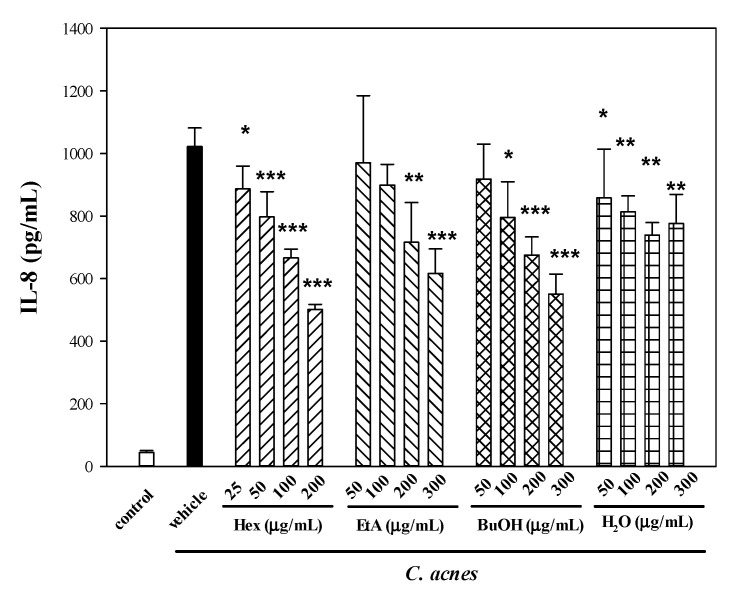
Effects of four partitioned fractions from ME of WBM leaf on *C. acnes*-induced IL-8 production in vitro. Four partitioned fractions as described in Figure 6, including *n*-hexane (Hex), ethyl acetate (EtA), *n*-butanol (*n*-BuOH) and water (H_2_O) sub-extracts. THP-1 cells were cultured with DMSO as the negative control, or co-incubated with *C. acnes* (M.O.I. = 75) and different concentrations of four respective sub-extracts for 24 h. IL-8 level was examined by the method described above. Each value shows the mean ± SD. Values with different symbols are significantly different from the *C. acnes* control (*C. acnes* alone) at *p* < 0.05 (*), *p* < 0.01 (**) and *p* < 0.001 (***).

In a separate study, to further inquire if four partitioned fractions from the ME of WBM leaf would suppress IL-8 production, the culture medium was supplemented with various concentrations (up to 800 μg/mL) of tested samples. There was no cytotoxic effect when THP-1 cells were incubated with culture medium containing as much as 200 μg/mL of the *n*-hexane (Hex), or 400 μg/mL of the other three sub-extracts, EtA, BuOH and H_2_O (data not shown). The production of IL-8 by *C. acnes*-stimulated THP-1 cells was markedly decreased by Hex (up to 51.2% at 200 μg/mL), followed by BuOH (46.6% at 300 μg/mL), EtA (40.0% at 300 μg/mL) and H_2_O (27.5% at 300 μg/mL) (Figure 2). Among the four sub-extracts, Hex exerted the most potent suppressive effect on IL-8 production.

Certain plant phytochemicals, such as polyphenols and flavonoids, can suppress the production of pro-inflammatory mediators, such as cytokines and eicosanoids, involved in the process of acute inflammation in humans [17]. In fact, one of the mechanisms underlying the anti-inflammatory effects of herbal extracts may be due, in part, to the antioxidant compounds they contain. It is widely recognized that excessive production of free radicals, such as hydroxyl, superoxide and nitrous oxide in sebum produced by *C. acnes*-infected sebaceous glands, and their uncontrolled regulation play a critical role in initiating and prolonging inflammation [18,19]. Phytochemicals exert their antioxidant properties by scavenging free radicals generated during the process of inflammation, thereby reducing oxidative stress and cell damage. We report that total phenolic contents of Hex, EtA, BuOH and H_2_O sub-extracts were 37.2 ± 2.42, 21.0 ± 0.76, 31.6 ± 1.22 and 26.6 ± 2.32 mg GAE/g, respectively. The presence of phytochemical compounds in the fractions may be partially responsible for the anti-inflammatory properties observed.

### 2.3. Effects of Ethanol-Soluble Fraction (ESF) on C. acnes-Induced Cellular IL-8 Production and Mouse Ear Edema

Since the Hex fraction from the ME of WBM leaf exerted the most potent suppressive effect on IL-8 production, it was therefore subjected to further separation techniques. Using silica gel column chromatography, three sub-fractions of the Hex fraction were collected. Among the three sub-fractions, the ethanol phase of the first sub-fraction (Hex-1) was cytotoxic at doses above 50 μg/mL. There was no cytotoxicity of Hex-3 at concentrations below 200 μg/mL, and no inhibitory effect on IL-8 production was observed (data not shown). The ethanol solution of the second sub-fraction (Hex-2) had no inhibitory effect on cell proliferation; however, Hex-2 had a significant suppressive effect on *C. acnes*-induced IL-8 production at concentrations below 100 μg/mL.

**Figure 3:**
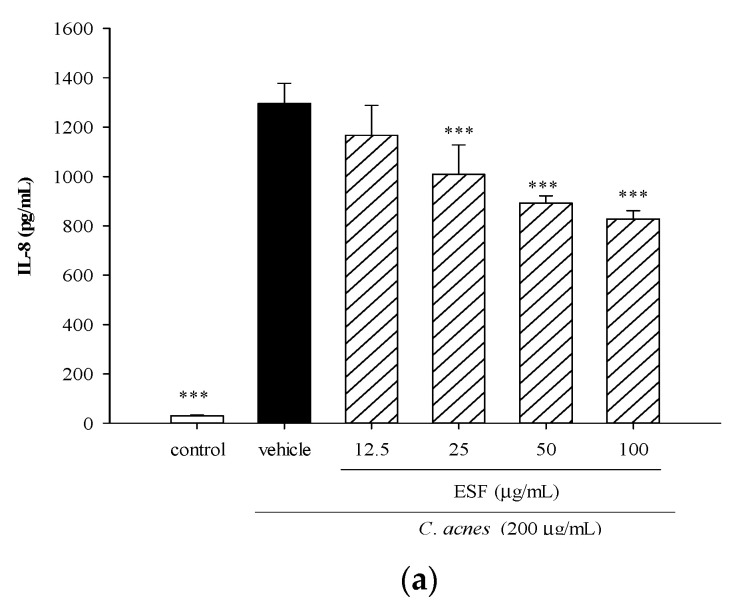

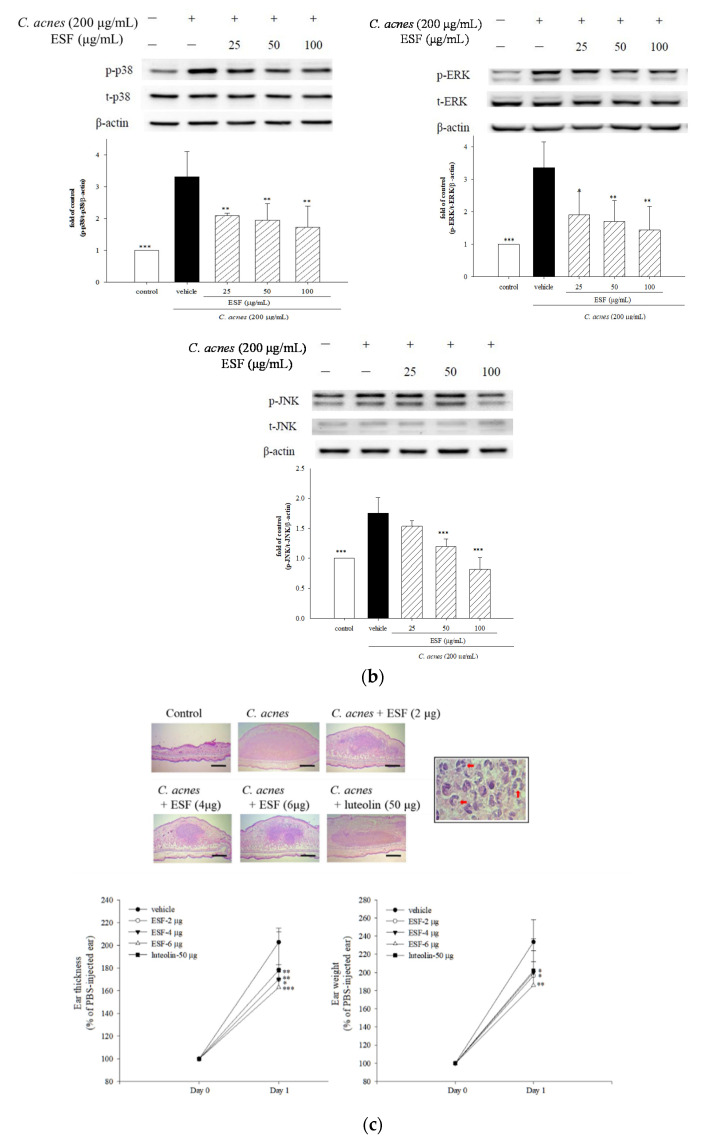
Effects of the ethanol-soluble fraction (ESF) of *n*-hexane layer on *C. acnes*-induced IL-8 production and mitogen-activated protein kinase (MAPK), p38, extracellular signal-regulated kinase (ERK) and c-Jun N-terminal kinase (JNK) activation in THP-1 cells and *C. acnes*-stimulated ear swelling in mice. THP-1 cells were cultured with DMSO as the negative control, or co-incubated with *C. acnes* (M.O.I. = 75) and different concentrations of ESF for 24 h (for IL-8 production) or 2 h (for MAPK activation). IL-8 concentration was determined using the method described above (**a**). MAPK activation was determined by western blot (**b**). In the ear edema mouse model, PBS, ESF (2, 4 or 6 μg/site) or luteolin (50 μg/site) was intradermally injected, immediately followed by the *C. acnes* injection. Infiltrated neutrophils were observed in a hematoxylin and eosin-stained cross section of the *C. acnes*-injected ear (×1000 magnification panel). Arrow (⟶): neutrophil infiltration. Scale bars represent 200 μm. The inhibitory effects of ESF and luteolin on *C. acnes*-stimulated mouse ear edema was quantified as described above (**c**). Each value shows the mean ± SD. Values with different symbols are significantly different from the *C. acnes* control (*C. acnes* alone) at *p* < 0.05 (*), *p* < 0.01 (**) and *p* < 0.001 (***).

We further explored the modulatory effect of different concentrations (12.5, 25, 50 or 100 μg/mL) of Hex-2 (designated as ESF) on *C. acnes*-induced IL-8 production, and we found that the ESF significantly lowered levels of IL-8 when the dose was 25 μg/mL or higher (Figure 3a), indicating that the ESF exerted in vitro anti-inflammatory effects in THP-1 monocytes. Since *C. acnes-*induced inflammatory responses are strongly regulated by the mitogen-activated protein kinase (MAPK) pathway [20], we inquire whether the ESF might be inhibiting IL-8 production by way of inactivating MAPK. Figure 3b shows that *C. acnes* stimulation significantly increased levels of phosphorylated p38, extracellular signal-regulated kinase (ERK) and c-Jun N-terminal kinase (JNK). However, incubation of *C. acnes*-stimulated THP-1 monocytes with the ESF significantly suppressed the expression of phosphorylated p38 by up to 48%, ERK by up to 43% and JNK by up to 46%.

Furthermore, using the mouse ear edema model, we observed that the ESF exerted in vivo anti-inflammatory properties by lowering *C. acnes*-stimulated ear thickness by up to 20% and ear biopsy weight by up to 23% (Figure 3c). The anti-inflammatory agent luteolin suppressed mouse ear swelling by 11.5%. We found a similar extent of mouse ear swelling relieved by the injection of the ESF (2–4 μg) or luteolin (50 μg), indicating that certain components in the ESF might account for the potent suppressive effect. This finding prompted us to identify which bioactive components might be responsible for the anti-inflammatory properties of the ESF sample.

**Figure 4:**
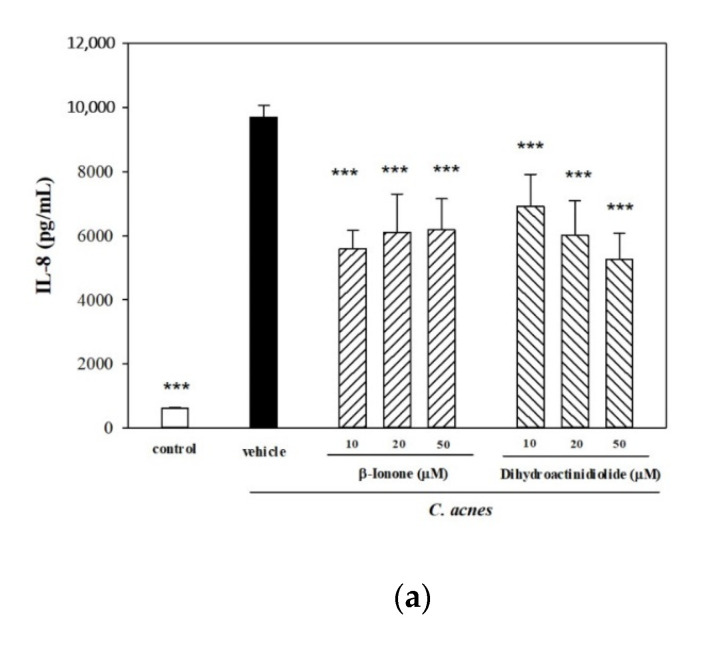

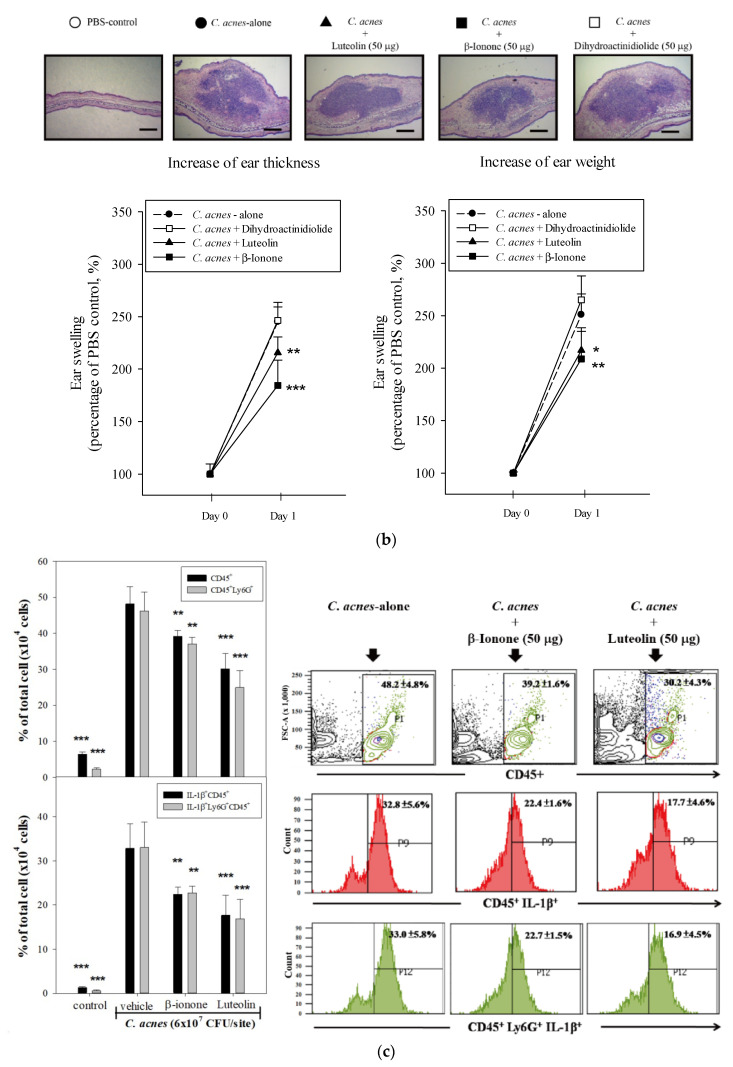
Effects of β-ionone or dihydroactinidiolide on *C. acnes*-induced inflammation in vitro and in vivo. THP-1 cells were co-incubated with *C. acnes* (M.O.I. = 75) and different concentrations (10, 20 or 50) of β-ionone or dihydroactinidiolide for 24 h. IL-8 level was examined by the method described above (**a**). PBS, β-ionone, dihydroactinidiolide or luteolin (50 μg/site) was intradermally injected, immediately followed by the *C. acnes* injection. Infiltrated neutrophils were observed in a hematoxylin and eosin-stained cross section of the *C. acnes*-injected ear. The inhibitory effects on *C. acnes*-stimulated mouse ear edema were evaluated by examined by the method described above (**b**). Evaluation of *C. acnes*-induced immune cells by flow cytometry in mouse ear after intradermal injection of PBS, β-ionone or luteolin. Twelve hours after the injection, flow cytometric analysis of the inflammatory cells harvested from *C. acnes*-induced ear tissues was performed. Cell suspensions were incubated with anti-CD45/PerCP and anti-Ly6G/FITC, and analyzed by flow cytometry (**c**). Each value shows the mean ± SD. Values with different symbols are considered to be significantly different from the *C. acnes* control (*C. acnes* alone) at *p* < 0.05 (*), *p* < 0.01 (**) and *p* < 0.001 (***).

MAPK, a family of serine/threonine protein kinases that includes p38, ERK and JNK, is involved in the regulation of a wide variety of fundamental cellular responses, physiologic functions and pathological processes [21]. It is widely accepted that the MAPK signaling cascade is activated by bacterial stimuli through TLR2, resulting in the over-expression and production of various pro-inflammatory mediators [22]. For example, MAPK plays a central role in *Streptococcus pneumoniae-* or *C. acnes*-stimulated inflammatory responses in murine microglia and human neonatal epidermal keratinocytes [20,22]. The results of the present study demonstrate that incubation of *C. acnes*-stimulated THP-1 cells with the ESF significantly attenuates MAPK activation, resulting in the suppression of IL-8 production (Figure 4). This finding corroborated our previous finding that expression of phosphorylated MAPK is suppressed by the unfractionated phenolic extract (TPE) of WBM leaf in THP-1 cells co-stimulated with *C. acnes* [14]. This fact might be strongly related to the cellular mechanisms underlying the ability of the ESF to lower the production of downstream pro-inflammatory mediators, thereby limiting in vivo inflammatory responses.

### 2.4. GC-MS Analysis of ESF

Using GC-MS, we separated and identified 24 known biological compounds in the ESF (Table 1). The identification and characterization of compounds were based on the order of elution in an HP-5MS column. The retention time, electron ionization mass spectrum (EIMS) fragments, molecular formula and the percentage of each of these compounds were also recorded. There were 11 fatty acid alkyl esters, 7 terpene-related compounds, 3 ketones, 2 alkanes and 1 aldehyde present in the ESF, and three major compounds were 3-phytylmenadione (vitamin K1) (15.8% of total), α-tocopherol (13.6%) and squalene (12.2%).

Previously, 3-phytylmenadione had been reported to suppress lipopolysaccharide (LPS)-stimulated IL-6 production by inactivating the nuclear factor kappa B (NF-κB) signaling pathway in murine RAW264.7 macrophages, human THP-1 monocytes and primary human fibroblasts [23,24]. Alpha-tocopherol exerted anti-inflammatory properties through the modulation of cell signaling cascades, such as protein kinase C (PKC), NF-κB and peroxisome proliferator-activated receptors (PPARs) [25]. Furthermore, the natural triterpene squalene suppressed production and expression of pro-inflammatory mediators by murine peritoneal macrophages and human monocytes and neutrophils [26]. Collectively, since these three bioactive components might account for the suppressive effect of the ESF on IL-8 production, they warrant further investigation.

In addition, methyl palmitate (9.9% of total), methyl linolenate (7%) and the other nine fatty acid alkyl esters (together 6.6%) were identified in the ESF sample. Methyl palmitate has been shown to reduce chemical-induced hepatotoxicity in rats [27], and inhibit phagocytosis and the release of pro-inflammatory mediators in primary rat Kupffer cells [28]. Methyl linolenate significantly blocks melanogenesis and inhibits tyrosinase activity in mouse B16 melanoma cells [29]. However, to date, there are no published reports of the anti-inflammatory properties of fatty acid acyl esters. In a separate work, we found that oleic acid, linoleic acid and α-linolenic acid cannot inhibit IL-8 production in *C. acnes*-stimulated THP-1 cells (data not shown).

Among non-nutrient components in the ESF fraction, no studies regarding bioactive properties and the inhibitory effect of five compounds (trans, trans-2,4-heptadienal, 6,10,14-trimethyl-2-pentadecanone, 4,8,12,16-tetramethylheptadecan-4-olide, *n*-pentacosane and *n*-heptacosane) on *C. acnes*-induced inflammatory responses have been conducted up to this point. Neophytadiene exerts a potent suppressive effect on LPS-induced inflammation in RAW264.7 macrophages [30], indicating this terpene compound might contribute, in part, to the suppressive effect of the ESF on *C. acnes*-stimulated IL-8 production.

Over time, we have become interested in knowing the anti-acne properties of non-nutrient phytochemicals in WBM. For example, we had previously reported that phytol, a precursor of vitamin E and vitamin K1, suppressed *C. acnes*-induced IL-8 production in THP-1 cells. [12]. In this study, β-ionone and dihydroactinidiolide were further identified by comparison of the recorded mass spectra with those of authentic standards (Supplementary Figure S1). Since β-ionone and dihydroactinidiolide are both precursors and metabolites of carotenoids, we inquired to determine whether anti-inflammatory properties of carotenoid-rich WBM extracts might be due, in part at least, to one or both of these compounds. Based on published reports, β-ionone is a metabolite of β-carotene and other carotenoids produced by enzymatic degradation [31]. It is also a precursor in the synthesis of vitamin A and β-carotene [32,33]. Recently, β-ionone has been reported to have vitamin A activity, exert anticarcinogenic and antitumor effects in human colon cancer cells [34], and reduce LPS-stimulated inflammation in murine BV-2 microglial cells [35]. Furthermore, dihydroactinidiolide, a structural analog of the anti-inflammatory agent loliolide, is a C11-terpene flavor compound present in a wide range of plants. A recent report demonstrated that dihydroactinidiolide exerted free radical scavenging activity with 1,1-diphenyl-2-picrylhydrazine (DPPH). It also has metal-chelating activity, and is neuroprotective against the Alzheimer’s amyloid β-peptide (Aβ_25–35_)-induced cytotoxicity in mouse neuroblastoma Neuro2A cells [36]. However, little is known about the effects of dihydroactinidiolide in immune cells. Thus, we further determined suppressive effect of β-ionone and dihydroactinidiolide on *C. acnes*-stimulated inflammation in THP-1 cells and mouse ear edema models.

### 2.5. Effects of β-Ionone and Dihydroactinidiolide on C. acnes-Induced Cellular IL-8 Production and Mouse Ear Edema

As shown in Figure 4a, β-ionone and dihydroactinidiolide significantly reduced IL-8 production (up to 46%) in *C. acnes*-stimulated THP-1 cells, and this suppressive effect on IL-8 production was comparable with that of the anti-inflammatory agent luteloin. When both compounds were tested to determine if they also exerted in vivo anti-inflammatory effect by reducing mouse ear edema, we found that a live culture of *C. acnes* stimulated the mouse ear to swell 2.5-fold (*p* < 0.001) and ear biopsy weight to increase 2.5-fold (*p* < 0.001) as compared to the untreated control (Figure 4b). Injection of β-ionone into mouse ears suppressed ear swelling by 24% (thickness) and 17% (weight), as well as the injection of luteloin by 12% (thickness) and 13% (weight) (Figure 4b). However, dihydroactinidiolide had no effect on ear thickness, but did slightly increase ear biopsy weight. These findings suggest that the anti-inflammatory properties of β-ionone and dihydroactinidiolide partially contributed to the suppressive effect of the ESF on IL-8 production in *C. acnes*-stimulated THP-1 cells. However, only β-ionone exerts an in vitro anti-inflammatory effect, and plays a critical role in the relief of mouse ear edema co-inoculated with the ESF and live *C. acnes*.

In a separate study, we inquired if β-ionone affected immune responses in *C. acnes*-stimulated mouse ear edema. The results of histological analysis demonstrated that inoculation of the mouse ear with live *C. acnes* causes epidermal microabscesses (Figure 4b). However, *C. acnes-*induced swelling was attenuated by β-ionone or luteolin treatment (Figure 4b). Flow cytometric analysis also showed that infiltration of leukocytes (CD45^+^) and neutrophils (CD45^+^Ly6G^+^) in mouse ear tissue was evident after 12 h of *C. acnes* stimulation (Figure 4c). Topical injection of β-ionone or luteolin along with the *C. acnes* administration significantly lowered the proportions of infiltrated inflammatory leukocytes (from 48% to 38%) and neutrophils (from 46% to 25–30%) and expression levels of inflammatory IL-1β in both cells approximately from 35% to 23% or 17% (Figure 4c).

A recent study by Fenini and colleagues demonstrated that increased amounts of IL-1β were released by activating inflammatory cells, keratinocytes and sebocytes with *C. acnes*. IL-1β and several other mediators markedly induced neutrophil recruitment to the skin [37], and infiltrated neutrophils produced large amounts of IL-1β to further promote the development of acne [38]. Since high levels of IL-1β were observed in *C. acnes*-induced human acne lesions and mouse skin lesions, Kistowska and colleagues suggested that IL-1β could be the critical role of *C. acnes* in the pathogenesis of acne vulgaris [38]. In the present study, β-ionone reduced immune cell migration and inflammatory cytokine gene expression, thereby decreasing epidermal microabscess and edema formation. We speculate that the in vivo anti-inflammatory effect of β-ionone on *C. acnes*-induced skin inflammation is attributable to the suppression of immune cell infiltration and IL-1β expression.

### 2.6. Effects of β-Ionone on Cellular IL-1β Production and Caspase-1 Expression

**Figure 5:**
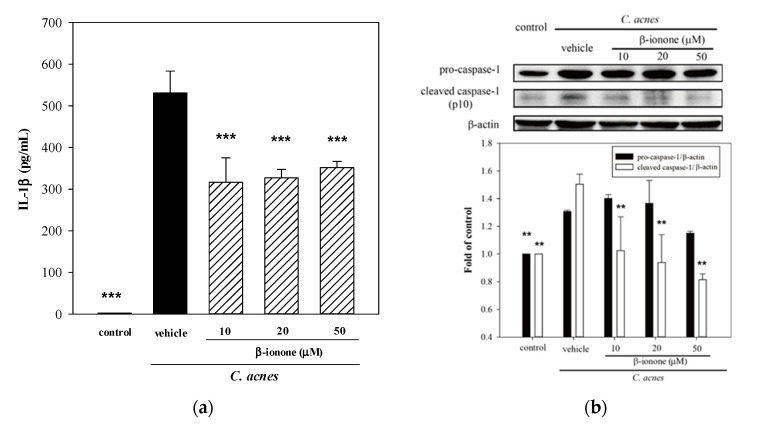
Effects of β-ionone on *C. acnes*-induced cellular IL-1β production and caspase-1 expression. THP-1 cells were cultured with DMSO as the negative control, or co-incubated with *C. acnes* (M.O.I. = 75) and different concentrations (10, 20 or 50 μM) of β-ionone for 24 h (IL-1β production) or 16 h (caspase-1 expression). The culture supernatants were subsequently collected and analyzed for IL-1β level (**a**). Caspase-1 expression was determined by western blot (**b**). Each value shows the mean ± SD. Values with different symbols are considered as significantly different from the *C. acnes* control (*C. acnes* alone) at *p* < 0.01 (**) and *p* < 0.001 (***).

Using human THP-1 monocytic cells, we investigated a possible mechanism underlying the inhibitory effect of β-ionone on *C. acnes*-stimulated IL-1β expression. Figure 5a shows that *C. acnes* significantly induced IL-1β production in THP-1 monocytes. The induction of IL-1β by *C. acnes* is considered as the major factor causing and prolonging skin inflammatory responses. When cells were co-incubated with *C. acnes* and β-ionone, levels of secreted IL-1β were significantly reduced by up to 39% (Figure 5a). We further inquired whether increasing production of IL-1β by *C. acnes* could be attributed to the induced expression of specific protease, caspase-1, previously called interleukin-1β converting enzyme (ICE), which is the rate-limiting enzyme involved in the cleavage pro-IL-1β (the precursor of IL-1β) to form IL-1β [39]. We observed that *C. acnes*-stimulated the over-expression of pro-caspase-1 (inactive zymogen of caspase-1) and proteolytic subunit p10 of pro-caspase-1 (active caspase-1) (Figure 5b). β-ionone did not affect expression of pro-caspase-1; however, it did significantly suppress cleaved caspase-1 by up to 54% (50 μM) as compared to the *C. acnes*-stimulated control (Figure 5b).

The data in Figure 4 and Figure 5 indicate that increasing levels of IL-1β expression and production in mouse ear edema and murine monocytes might be due, in part, to the stimulation of active caspase-1 expression by live *C. acnes*. Our findings were in accordance with results of previous reports which show that *C. acnes*-upregulated caspase-1 gene expression triggers the cleavage of pro-IL-1β to promote the production and secretion of IL-1β [40,41]. It is widely recognized that caspase-1 is a critical regulator of the inflammatory response, and that his highly specific protease is activated by a cytosolic multi-protein complex, named inflammasome (caspase-1-activating platform), in response to exposure to *C. acnes*. Since excessive levels of IL-1β, caspase-1 and NLRP3 inflammasome were detected in *C. acnes*-infected pilosebaceous follicles and skin lesions, advancing the understanding of the molecular mechanisms regarding IL-1β, caspase-1 and inflammasome may provide knowledge into acne pathogenesis that might identify targets for the therapy of acne.

Using the bioassay-guided isolation approach, we herein demonstrated that the ME of WBM leaf and its four partitioned fractions suppressed *C. acnes*-induced inflammatory responses in THP-1 cells. Furthermore, the ESF collected from the most potent hexane fraction significantly reduced *C. acnes*-induced cellular IL-8 production through the inactivation of MAPK cell signaling, and alleviated *C. acnes*-stimulated mouse ear edema. Our results show that the ESF is more potent than luteolin, an anti-inflammatory agent, in suppressing *C. acnes*-induced inflammation. We therefore suggest that this ESF should be considered as a therapeutic means to alleviate or relieve inflammatory skin diseases. Future in vivo investigations of the anti-inflammatory, toxicological and physiological aspects of the effects of the ESF on skin inflammation are warranted.

In addition to WBM leaf extracts, β-ionone significantly reduced *C. acnes*-stimulated pro-inflammatory IL-1β or IL-8 production and mouse ear edema. The anti-inflammatory effect of β-ionone on immune responses might be due, in part, to the suppression of *C. acnes*-induced upregulated caspase-1 gene expression. A limitation of the present study is that β-ionone was not one of major compounds among 24 identified components of the ESF, and our findings accounted for the partial anti-inflammatory properties of the ESF we observed. Additional studies are needed to further determine key components involving in the suppressive effects of the ESF on inflammatory responses.

In conclusion, we have demonstrated that methanolic extracts of WBM leaf, four partitioned fractions from the ME and the ESF were prepared using bioassay-guided isolation techniques. The ESF significantly suppresses *C. acnes*-stimulated MAPK-mediated pro-inflammatory IL-8 production in human monocytes, and reduces ear swelling in a *C. acnes*-induced mouse ear edema model. Furthermore, β-ionone from the ESF lowered caspase-1 over-expression and pro-inflammatory mediator production in THP-1 monocytes, and lessened epidermal microabscess, neutrophilic infiltration and IL-1β expression in the mouse ear. Collectively, our findings supported that WBM leaf extracts exerted anti-inflammatory properties, and also demonstrated that the ESF is a potential anti-inflammatory agent for modulating in vitro and in vivo inflammatory responses.

## 3. Materials and Methods

### 3.1. Materials

3-(4,5-dimethylthiazol-2-yl)-2,5-diphenyltetrazolium bromide (MTT), β-ionone, dimethylsulfoxide (DMSO), luteolin and sodium dodecyl sulfate (SDS) were purchased from Sigma Chemical Co. (St. Louis, MO, USA). Dihydroactinidiolide was purchased from AK Scientific, Inc (Union, CA, USA). Brain heart infusion (BHI) broth was obtained from Difco (Detroit, MI, USA). Fetal bovine serum (FBS), phosphate-buffered saline (PBS), RPMI1640 medium, penicillin and streptomycin were from Gibco (Carlsbad, CA, USA). The ELISA assay kits for detecting interleukin (IL)-1β or IL-8 were obtained from Invitrogen (Carlsbad, CA, USA). All reagent-grade organic solvents were purchased from Burdick and Jackson (Muskegon, MI, USA).

### 3.2. Isolation and Determination of Active Compounds from WBM Leaf Extract

**Figure 6:**
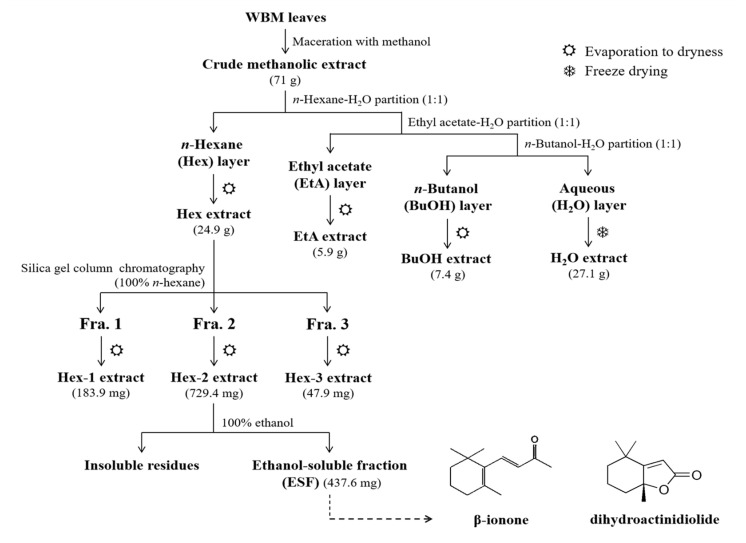
Flowchart of the extraction methods used to separate the anti-inflammatory components from WBM leaf. Fractions were designated as Hex-1, Hex-2 and Hex-3, as indicated in the figure. The structures of 1 (β-ionone) and 2 (dihydroactinidiolide) isolated from the Hex-2 layer.

Wild bitter melon leaves (Hualien No.1) were obtained from the Hualien District Agricultural Research and Extension Station (Hualien, Taiwan). The leaves were washed, air dried and then finely ground and extracted with methanol. As shown in Figure 6, ground WBM leaf powder was immersed and extracted with methanol (1:20, *w*/*v*) at room temperature for 4 h. The residue was re-extracted overnight with methanol (1:20, *w*/*v*). The combined filtrates were then centrifuged at 12,000× *g* for 10 min, and evaporated to dryness to yield the ME (19.1% of the original weight). Next, combined MEs of WBM leaf (71 g) were dissolved and suspended in 200 mL of water in a separatory funnel prior to being partitioning sequentially with *n*-hexane, ethyl acetate and *n*-butanol (200 mL each for one time). The yield of fractionated extracts was based on the weight of the crude methanol extract. Under reduced pressure evaporation or freeze drying, the four sub-extract weights were as follows: *n*-hexane (Hex) (24.9 g, 35% of yield), ethyl acetate (EtA) (5.90 g, 8.3% of yield), *n*-butanol (BuOH) (7.35 g, 10.3% of yield) and aqueous solution (H_2_O) (27.1 g, 38.2% of yield). All extracts were stored frozen at −20 °C until used. Following bioassay-guided procedures, the active sub-extract Hex was further fractionated using chromatographic techniques. Hex (10 g) was then re-dissolved with *n*-hexane and separated using silica gel column chromatography (Silicycle SiliaFlash P60 230–400 mesh). Three fractions were collected and evaporated to dryness to give “Hex-1” (184 mg; yellow), “Hex-2” (729 mg; brownish-red) and “Hex-3” (47.9 mg; light yellow). The three sub-extracts were dissolved in ethanol. Hex-3 was totally dissolved in ethanol, however, Hex-1 and Hex-2 were dissolved in ethanol, resulting in the insoluble residue and ethanol-soluble fraction. The ethanol solution of Hex-2 was then evaporated under reduced pressure and designated the “ethanol-soluble fraction (ESF)” extract (438 mg).

The ME of WBM leaf and the various fractions of the *n-*hexane layer were weighed and re-constituted with a known volume of DMSO for use in the subsequent experiments in *C. acnes*-stimulated THP-1 cells. Their inhibitory effects against *C. acnes*-induced inflammatory responses were then determined.

### 3.3. Analysis of ESF by Gas Chromatography–Mass Spectrometry

The ESF from the *n*-hexane layer was analyzed by gas chromatography (Agilent Technologies, Palo Alto, CA, USA) and mass spectrometry using electron impact ionization mode, quadruple mass filter and a fused silica capillary column (HP-5MS; 30 m × 0.25 mm, i.d., film thickness 0.25 μm, Agilent, Santa Clara, CA, USA). Helium was the carrier gas. The injection port and detector temperatures were set at 250 and 280 °C, respectively. The oven temperature program was set at 40 °C, then increased to 280 °C at 6.5 °C per min, and held at 280 °C for 34 min. Mass spectrometry (MS) conditions were at an ionization voltage of 70 eV with a scan range of 40–500 Da (scan rate = 1 scan/s). The mass spectra of the substances in the ESF were compared with those of standards in the database NIST02.L and Wiley7n.1 mass spectra libraries. The validity of key data was checked using authentic standards such as β-ionone and dihydroactinidiolide (Figure 6 and Supplementary Figure S1).

### 3.4. Bacterial and Cell Cultures and Growth Conditions

A *C. acnes* strain (BCRC10723) and human monocytic leukemia (THP-1) cell line (BCRC60430) were obtained from the Bioresource Collection and Research Center (Hsinchu, Taiwan). *C. acnes* was cultured in BHI broth with 1% (*w*/*v*) glucose in an anaerobic atmosphere using the BBL GasPak system (Becton Dickinson, Cockeysville, MD, USA). THP-1 cells were maintained in RPMI1640 medium supplemented with 10% (*v*/*v*) heat-inactivated FBS, penicillin (100 U/mL) and streptomycin (100 µg/mL) at 37 °C in a humidified environment with 5% CO_2_. To monitor cell viability, the method of AlamarBlue^®^ (Invitrogen, Carlsbad, CA, USA) was used.

### 3.5. Measurement of IL-8 Production in Human Monocytic THP-1 Cells

The cellular assay techniques that employ live *C. acnes*-infected cells, including human osteoblasts, THP-1 monocytes and peripheral blood mononuclear cell-derived monocytes, have been reported [14,36,42]. A well-established co-culture model of *C. acnes* and human THP-1 monocytes was used to quantify the suppressive effects of WBM extracts and their constituents on *C. acnes*-stimulated IL-8 production [14]. A culture of *C. acnes* was incubated to reach the logarithmic phase, and bacterial pellets were harvested by centrifugation at 10,000× *g* for 5 min, washed thrice with PBS and resuspended in RPMI cell culture medium. THP-1 cells were planted into 24-well plates with serum-free RPMI medium at a density of 1 × 10^6^ cells/mL per well, and then stimulated with live *C. acnes* (multiplicity of infection (M.O.I.) = 75) alone or in combination with tested samples at 37 °C in a 5% CO_2_ humidified atmosphere. After 24 h of incubation, the cell-free culture supernatants were collected, and the levels of IL-1β or IL-8 were determined using the corresponding enzyme immunoassay kits.

### 3.6. Protein Lysate Preparation and Western Blot Analysis

Human THP-1 cells (2 × 10^6^ cells/mL) were seeded in 6 cm dishes, and stimulated with viable *C. acnes* (M.O.I. = 75) alone or co-incubated with various concentrations of the ESF (25, 50 or 100 μg/mL) or β-ionone (10, 20 or 50 μM). After 2 h or 16 h of treatment, cells were harvested and washed with PBS. Whole cell lysates were prepared in lysis buffer (Cell Signaling, Beverly, MA, USA) containing 10 mM phenylmethylsulfonyl fluoride (PMSF). Cell lysates were sonicated and clarified by centrifugation at 4 °C, 12,000 g for 10 min. The concentration of total cellular protein was determined using the detergent compatible (DC) rotein assay (Bio Rad, Hercules, CA, USA). Heat-denatured cellular protein samples (30 μg) were separated electrophoretically using 10% (*w*/*v*) SDS-polyacrylamide gel (SDS-PAGE), and subsequent transfer to polyvinylidene difluoride (PVDF) membranes (Millipore, MA, USA). Membranes were blocked by incubation in gelatin-NET buffer containing 1% (*w*/*v*) bovine serum albumin at room temperature and subsequently incubated with an appropriate dilution of primary antibodies of mitogen-activated protein kinase (MAPK), phospho-MAPK, pro-caspase-1, cleaved caspase-1 (Cell Signaling Technology, Danvers, MA, USA) or β-actin (Sigma) overnight at 4 °C, followed by reacting with a secondary antibody (1:5000 dilution of immunoglobulin conjugated with horseradish peroxidase (Sigma)). The immuno-reactive proteins if interest were developed and detected with the enhanced **ECL**™ chemiluminescence western blotting detection system (ChemiDoc XRS, Bio-Rad, Hercules, CA, USA). Signal strengths were quantified using a densitometric program (Image Lab, Bio-Rad).

### 3.7. C. acnes-Induced Inflammation in Mouse Ears

Eight-week-old male Institute of Cancer Research (ICR) ICR mice were purchased from the Animal Center of the College of Medicine, National Taiwan University (Taipei, Taiwan). Protocols involving animals were approved by the Institutional Animal Care and Use Committee of the National Taiwan Normal University. Using the modified Hsu’s method previously described [12], we evaluated the in vivo anti-inflammatory properties of samples of interest in a *C. acnes*-stimulated mouse ear edema model. *C. acnes* (6 × 10^7^ CFU per 10 μL in PBS) was intradermally injected into the right ear of mice, while the left ears received an equal volume (10 μL) of PBS (*n* = 5) as a control. Ten microliters of tested samples in 5% DMSO in PBS were injected into the same site of both ears immediately following *C. acnes* or PBS injection (*n* = 6). Preliminary studies informed us of the doses of ME (0.5 mg/10 μL/site), ESF (6 μg/10 μL/site), β-ionone (6 μg/10 μL/site), dihydroactinidiolide (50 μg/10 μL/site) or luteolin (50 μg/10 μL/site) that are optimal for topical testing without evident skin irritation (data not shown). Luteolin was applied as a control herein. Twenty-four hours after injection, ear thickness was measured using a micro-caliper (Mitutoyo, Kanagawa, Japan). Mice were then sacrificed by means of carbon dioxide asphyxiation, and punched ear disks (4.0 mm) were collected and weighed. The extent of edema in each mouse was evaluated according to results of differences in thickness and weight between both ears. The increase in ear thickness and weight of the *C. acnes*-stimulated ear was calculated and expressed as a percentage of the PBS control. For histological examination, mouse ears were embedded in paraffin, cut vertically into cross sections, stained with hematoxylin and eosin (H&E) and then viewed under a microscope for the evaluation of inflammatory response.

### 3.8. Flow Cytometric Analysis of Single-Cell Suspensions Prepared from Skin

In a separate study, ears of mice were intradermally injected with *C. acnes* alone, *C. acnes*/β-ionone or *C. acnes*/luteolin, as described above. Twelve hours later, the ears were excised (*n* = 5), split into dorsal and ventral halves, and then dispersed by passage through a 70 μm cell strainer (BD Biosciences) into the RPMI medium. Each sample was then brought to a final volume of 4 mL with RPMI medium. Cells were washed with PBS and filtered again through a 40 μm cell strainer (BD Biosciences). Single-cell suspensions were incubated with fluorescein isothiocyanate (FITC)-conjugated anti-mouse Ly6G, a neutrophil marker (BioLegend, San Diego, CA, USA), and peridinin chlorophyll protein (PerCP)-conjugated anti-mouse CD45, a common leukocyte marker (BioLegend), in fluorescence-activated cell sorting (FACS) buffer (PBS containing 0.5% *w*/*v* bovine serum albumin and 0.09% sodium azide) for 30 min and washed three times with FACS buffer. For intracellular cytokine analysis, cells were washed, surface stained as described above, fixed and permeabilized for intracellular staining of allophycocyanin (APC)-conjugated anti-IL-1β (eBioscience, San Diego, CA, USA), as instructed by the manufacturer. Samples were analyzed with FacsCantoII (BD Biosciences) using FACS Diva software, and the data were analyzed using FlowJo software.

### 3.9. Statistical Analysis

All data were calculated and presented as means ± SD. Statistical analyses were performed with SPSS Statistics for Windows, version 22.0 (SPSS Inc., Chicago, IL, USA). Statistical significance was determined using one-way ANOVA followed by the least significance difference (LSD) multiple range test. Mean differences among groups were considered statistically significant at the *p* ≤ 0.05 levels.

## Figures and Tables

**Table 1:** Relevant MS data of the compounds identified from ESF (EI, 70 eV).

No.	RT (min)	Identification	EIMS Fragments (70 eV), *m*/*z* (Relative Abundance)	Molecular Formula	% of Total
1	7.8	*trans*, *trans*-2,4-heptadienal	110 (M+, 25), 81 (100), 68 (14), 53 (20), 41 (20)	C_7_H_10_O	1.3
2	18.25	β-ionone	192 (M+, 4), 177 (100), 135 (14), 123 (100), 43 (45)	C_13_H_20_O	1.3
3	19.14	dihydroactinidiolide	180 (M+, 24), 137 (42), 111 (100), 67 (23), 43 (35)	C_11_H_16_O_2_	2.1
4	19.80	*cis*-3-hexenyl benzoate	105 (100), 82 (83), 77 (44), 67 (56), 51 (10)	C_13_H_16_O_2_	1.7
5	24.24	neophytadiene	278 (M+, 3), 95 (89), 82 (80), 68 (100), 57 (69)	C_20_H_38_	1.5
6	24.34	6,10,14-trimethyl-2-pentadecanone	109 (28), 85 (35), 71 (53), 58 (97), 43 (100)	C_18_H_36_O	0.9
7	24.91	phytol	123 (63), 95 (88), 82 (100), 68 (72), 57 (66)	C_20_H_40_O	0.6
8	25.26	palmitoleic acid, methyl ester	268 (M+, 4), 236 (21), 83 (51), 69 (73), 55 (100)	C_17_H_32_O_2_	0.3
9	25.56	palmitic acid, methyl ester	270 (M+, 12), 227 (15), 143 (21), 87 (69), 74 (100)	C_17_H_34_O_2_	9.9
10	26.58	palmitic acid, ethyl ester	284 (M+, 12), 101 (52), 88 (100), 73 (19), 55 (28)	C_18_H_36_O_2_	0.2
11	28.05	linoleic acid, methyl ester	294 (M+, 18), 95 (72), 81 (100), 67 (100), 55 (64)	C_19_H_34_O_2_	0.8
12	28.16	linolenic acid, methyl ester	292 (M+, 6), 108 (43), 95 (59), 79 (100), 67 (61)	C_19_H_32_O_2_	7
13	28.48	stearic acid, methyl ester	298 (M+, 16), 143 (22), 87 (69), 74 (100), 43 (28)	C_19_H_38_O_2_	1.5
14	31.15	eicosanoic acid, methyl ester	326 (M+, 25), 87 (77), 74 (100), 55 (51), 43 (54)	C_21_H_42_O_2_	0.5
15	31.50	4,8,12,16-tetramethylheptadecan-4-olide	126 (14), 99 (100), 83 (24), 69 (25), 43 (30)	C_21_H_40_O_2_	0.2
16	31.80	dodecanoic acid, 2-hexen-l-yl ester	82 (100), 67 (23), 55 (18), 43 (15)	C_18_H_34_O_2_	0.9
17	33.23	*n*-pentacosane	352 (M+, 3), 85 (56), 71 (77), 57 (100), 43 (56)	C_25_H_52_	0.3
18	33.60	docosanoic acid, methyl ester	354 (M+, 30), 143 (24), 87 (76), 74 (100), 43 (37)	C_23_H_46_O_2_	0.4
19	35.52	*n*-heptacosane	380 (M+,3), 85 (56), 71 (83), 57 (100), 43 (56)	C_27_H_56_	0.4
20	35.89	tetracosanoic acid, methyl ester	382 (M+, 34), 143 (31), 87 (70), 74 (100), 43 (47)	C_25_H_50_O_2_	0.3
21	37.01	squalene	410 (M+, 1), 95 (15), 81 (58), 69 (100), 41 (20)	C_30_H_50_	12.2
22	39.89	γ-tocopherol	416 (M+, 90), 191 (18), 151 (100)	C_28_H_48_O_2_	2.6
23	41.30	α-tocopherol	430(M+, 100), 205 (10), 165 (100)	C_29_H_50_O_2_	13.6
24	45.77	3-phytylmenadione (vitamin K1)	450(M+, 100), 225 (58), 198 (44), 186 (52)	C_31_H_46_O_2_	15.8

## Supplementary Materials

The following are available online. Figure S1: Gas chromatography–mass spectrometry (GC-MS) fragmentation patterns of β-ionone and dihydroactinidiolide.
