# Supplementary material for: In Vitro and In Vivo Screening of Wild Bitter Melon Leaf for Anti-Inflammatory Activity against Cutibacterium acnes
Source: Molecules. 2020 Sep 18;25(18):4277. doi: 10.3390/molecules25184277 (PMC7570529; doi:10.3390/molecules25184277)
Supplement: Supplementary file 1 [file molecules-25-04277-s001.pdf]

In Vitro and In Vivo Screening of Wild Bitter Melon Leaf for Anti-Inflammatory Activity against *Cutibacterium acnes*

Lu-Te Chuang [1], Ya-Hsin Shih [2], Wen-Cheng Huang [2], Lie-Chwen Lin [3], Chin Hsu [4], Jong-Ho Chyuan [5], Tsung-Hsien Tsai [6,*] and Po-Jung Tsai [2,7,*]

1. Department of Biotechnology and Pharmaceutical Technology, Yuanpei University of Medical Technology, Hsinchu 3, Taiwan; ltchuang@mail.ypu.edu.tw
2. Department of Human Development and Family Studies, National Taiwan Normal University, Taipei 1, Taiwan; yayayungchieh@hotmail.com (Y.-H. S.); wencheng7373@gmail.com (W.-C. H.)
3. National Research Institute of Chinese Medicine, Ministry of Health and Welfare, Taipei 1, Taiwan; lclin@nricm.edu.tw
4. Department of Exercise Health Science, National Taiwan University of Sport, Taichung 4, Taiwan; jeanhsu@ntupes.edu.tw
5. Hualien District Agricultural Research and Extension Station, Hualien 9, Taiwan; jonghoc@hdares.gov.tw
6. Department of Dermatology, Taipei Municipal Wan Fang Hospital and Taipei Medical University, Taipei 1, Taiwan
7. Program of Nutrition Science, School of Life Science, National Taiwan Normal University, Taipei 1, Taiwan

* Correspondence: thtsai2@yahoo.com.tw (T.-H. T.); pjtsai@ntnu.edu.tw (P.-J. T.); Tel.: +886-2-7749-1455 (P.-J. T.)

Supplementary Materials: The following are available online at www.mdpi.com/xxx/s1, Figure S1: Gas chromatography–mass spectrometry (GC-MS) fragmentation patterns of β-ionone and dihydroactinidiolide.

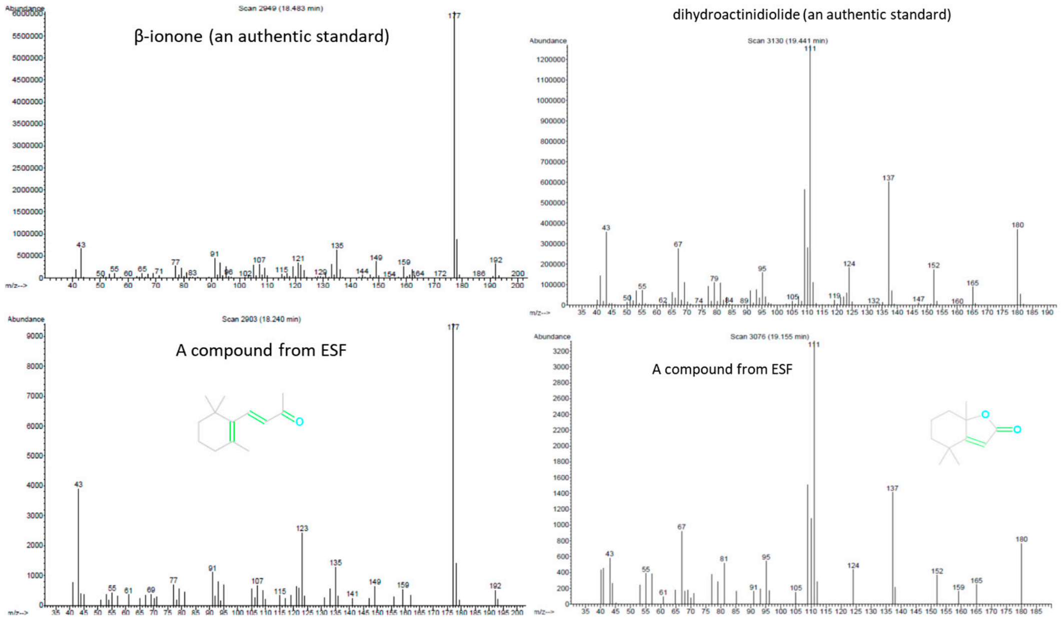

(a) (b)

Supplementary Figure 1. Gas chromatography–mass spectrometry (GC-MS) fragmentation patterns of β-ionone (**a**) and dihydroactinidiolide (**b**). The validity of two compounds was examined using respective authentic standards.
